# Comparison of Risk of Osteoporotic Fracture in Denosumab vs Alendronate Treatment Within 3 Years of Initiation

**DOI:** 10.1001/jamanetworkopen.2019.2416

**Published:** 2019-04-19

**Authors:** Alma B. Pedersen, Uffe Heide-Jørgensen, Henrik T. Sørensen, Daniel Prieto-Alhambra, Vera Ehrenstein

**Affiliations:** 1 Department of Clinical Epidemiology, Aarhus University Hospital, Aarhus, Denmark; 2 Pharmaco- and Device Epidemiology, Centre for Statistics in Medicine, Oxford NIHR Musculoskeletal Biomedical Research Unit, Nuffield Department of Orthopaedics, Rheumatology, and Musculoskeletal Sciences, University of Oxford, Oxford, United Kingdom; 3 Grup de Recerca en Epidemiologia de las Malalties Prevalents de l’Aparell Locomotor Research Group, Centro de Investigación Biomédica en Red Fragilidad y Envejcimiento Saludable (CIBERFes), Instituto de Salud Carlos III and Universitat Autonoma de Barcelona, Barcelona, Spain

## Abstract

**Importance:**

Head-to-head randomized clinical trials showed greater efficacy of denosumab vs alendronate in improving bone mineral density. Although there is an association of changes in bone mineral density with reductions in fracture risk, the magnitude of the association is not well established.

**Objective:**

To compare the risk of hip and any fracture in patients treated with denosumab and alendronate in routine practice settings.

**Design, Setting, and Participants:**

This Danish nationwide, population-based, historical cohort study of a population with universal access to health care used prospectively collected, individually linked data from Danish health registries with complete follow-up. Cohorts consisted of 92 355 individuals 50 years or older who were new users of denosumab (n = 4624) or alendronate (n = 87 731) from May 2010 to December 2017 after at least 1 year without an antiosteoporosis medication dispensing.

**Exposures:**

Initiation of denosumab or alendronate.

**Main Outcomes and Measures:**

The primary outcome was hospitalization for hip fracture, and the secondary outcome was hospitalization for any fracture. Inverse probability of treatment weights and the intention-to-treat approach were used to calculate cumulative incidences and adjusted hazard ratios (aHRs) with 95% CIs.

**Results:**

Of the 92 355 included patients, 75 046 (81.3%) were women, and the mean (SD) age was 71 (10) years. The denosumab cohort had a lower proportion of men than the alendronate cohort (12.7% [589] vs 19.0% [16 700]), while age distributions were similar in the 2 cohorts. Within 3 years of follow-up, initiation of denosumab or alendronate was associated with cumulative incidences of 3.7% and 3.1%, respectively, for hip fracture and 9.0% and 9.0%, respectively, for any fracture. Overall, the aHRs for denosumab vs alendronate were 1.08 (95% CI, 0.92-1.28) for hip fracture and 0.92 (95% CI, 0.83-1.02) for any fracture. The aHR of denosumab vs alendronate for hip fracture was 1.07 (95% CI, 0.85-1.34) among patients with a history of any fracture and 1.05 (95% CI, 0.83-1.32) among patients without history of fracture. The aHR for any fracture for denosumab vs alendronate was 0.84 (95% CI, 0.71-0.98) among patients with a history of any fracture and 0.77 (95% CI, 0.64-0.93) among patients with no history of fracture.

**Conclusions and Relevance:**

Treatment with denosumab and alendronate was associated with similar risks of hip or any fracture over a 3-year period, regardless of fracture history.

## Key Points

**Question:**

What is the real-world risk of fracture for patients treated with denosumab compared with those treated with alendronate?

**Findings:**

In this Danish cohort study including 4624 individuals treated with denosumab and 87 731 individuals treated with alendronate, 3-year cumulative incidence of hip fracture was 3.7% and 3.1% among the denosumab and alendronate cohorts, respectively. The 3-year cumulative incidence of any fracture was 9.0% for both cohorts.

**Meaning:**

In routine clinical practice, initiation of denosumab and alendronate treatments were associated with similar risks of hip and any fracture over a 3-year period.

## Teaser

This cohort study of individually linked data from Danish health registries compares the risk of hip and any fracture in adult patients treated with denosumab vs alendronate in routine practice settings.

## Introduction

Osteoporosis is characterized by progressive deterioration of bone structure and decreased bone mineral density (BMD).^1^ Fractures are a common manifestation of osteoporosis,^2,3^ with hip fracture being the most serious.^4,5^ Worldwide, osteoporosis is the most common metabolic skeletal disease,^6^ affecting 200 million persons and causing nearly 9 million fractures annually.^2^

Bisphosphonates are a mainstay of prevention and treatment of postmenopausal osteoporosis, with alendronate being the first-line choice, given its effectiveness and low price.^7,8^ Denosumab was approved in the European Union, including Denmark, in May 2010 for the treatment of osteoporosis in postmenopausal women and in men at high fracture risk.^9^ Both denosumab and alendronate are antiresorptive drugs and inhibit osteoclasts, albeit via different mechanisms; while denosumab binds the cytokine receptor activator of nuclear factor–κB ligand and thereby blocks osteoclasts’ formation, maturation, activation, and survival, alendronate binds to bone mineral, where it is absorbed by mature osteoclasts, inducing osteoclast apoptosis and suppressing resorption.^10^

Some adverse effects reported among bisphosphonates users, such as gastrointestinal tract effects or acute kidney injury, are rarely seen with denosumab, owing to differences in administration and clearance between the 2 agents.^11^ In contrast to weekly oral alendronate treatment, denosumab treatment, administered subcutaneously every 6 months, removes the need for patient adherence for that period.^12^ On the other hand, denosumab is promptly cleared after 6 months, while bisphosphonates linger in bone for many years, potentially partially compensating for suboptimal adherence. Denosumab is a cost-effective treatment for osteoporosis with €50 000 to €60 000 (US$56 232-$67 478) per quality-adjusted life-year gained^13^ and is a potential alternative to alendronate in patients 75 years or older, those with renal impairment, and those with low expected adherence.^14^

In randomized clinical trials (RCTs), denosumab was more efficacious than bisphosphonates in increasing bone mass among postmenopausal women.^15,16^ Although there is an association of change in BMD with reduction in fracture risk,^17,18^ the magnitude of the association is not well established. The RCTs examined the risk of any fracture as a secondary outcome, finding no difference in the fracture risk between denosumab and alendronate within 1 year of treatment initiation.^15,16^ However, these studies were based on a low number of fractures, and the extent of fracture reduction with denosumab compared with alendronate in routine clinical practice is unclear from the data. Using routinely collected data from population-based health registries in Denmark, we conducted a nationwide cohort study to compare risk of hip and any fracture in patients treated with denosumab and alendronate.

## Methods

### Setting and Data Sources

Danish residents have access to universal health care, including subsidized prescription costs. Furthermore, numerous population-based linkable registries enable nearly complete capture of important life and health events. To construct the analysis data set for this study, we linked data from the Danish Civil Registration System, which assigns a unique personal identifier to all Danish residents and tracks vital status and migration^19^; the Danish National Health Services Prescription Database, which has recorded reimbursed medication dispensings from all community pharmacies since 2004^20^; and the Danish National Patient Registry, which contains discharge dates and diagnoses from all hospitalizations since 1977 and from all outpatient clinic and emergency department contacts since 1995.^21^ Although dispensings are imperfect representations of true treatment status, they are considered a better measure of medication intake compared with most alternatives.^22^ Completeness and positive predictive values of the fracture diagnoses in the Danish National Patient Register exceed 90%,^23,24^ and positive predictive value of the comorbidities is 90%.^25^

As this cohort study did not involve contact with patients or an intervention, it was not necessary to obtain permission from the Danish Scientific Ethical Committee. The study was approved by the Danish Data Protection Agency. Danish law does not require informed consent or ethical approval for studies based solely on registry data. This report follows the Strengthening the Reporting of Observational Studies in Epidemiology (STROBE) reporting guideline for cohort studies.

### Patients

We used an active-comparator, new-user approach^26^ to include patients taking denosumab (the denosumab cohort) and patients taking alendronate (the alendronate cohort), defined as persons with the first dispensing of denosumab or alendronate between May 26, 2010, and December 31, 2017. The date of the first dispensing of denosumab or alendronate during the study period was the index date in each respective cohort. We excluded patients with a recorded dispensing of any osteoporosis medication (eg, denosumab, alendronate, raloxifene, ipriflavone, strontium ranelate, other bisphosphonates, teriparatide, calcitonin) in the 12 months preceding the index date. We furthermore excluded patients younger than 50 years on the index date and those with a history of cancer or Paget disease in the 10 years before index date (both of which are alternative indications for denosumab and alendronate).

### Outcome

The primary outcome was hip fracture, identified by primary or secondary diagnosis during hospitalization. The secondary outcome was any fracture, including primary or secondary diagnosis during hospitalization for hip or vertebral fractures and primary or secondary diagnosis for nonvertebral nonhip fractures recorded during any hospital encounter (inpatient, outpatient, or emergency).

### Covariates

We measured the following covariates at the index date: age, sex, and comorbidities, including history of fracture, alcohol-related disorders, obesity, and diseases from the Charlson Comorbidity Index (CCI). We summarized diseases from the CCI using the Romano modification,^27^ ie, excluding chronic obstructive pulmonary disease (a marker of smoking) and chronic renal impairment (a predictor of denosumab treatment), which were treated as separate covariates. We classified the CCI scores as no comorbidity (CCI score, 0), moderate comorbidity (CCI score, 1-2), or severe comorbidity (CCI score, ≥3). We also included data on dispensings of comedications affecting bone metabolism or fracture risk since 2004. Included comedications were oral corticosteroids, anticoagulants and antithrombotics, hormone replacement therapy, anxiolytics and sedatives, antipsychotics, antidepressants, statins, nonsteroid anti-inflammatory drugs, antihypertensive drugs, drugs for treatment of chronic obstructive pulmonary disease, opioids, and antithyroid drugs. History of hip and any fracture was defined using the same codes as those used to define hip and any fracture outcome.

### Statistical Analysis

We tabulated the characteristics of the denosumab cohort and the alendronate cohort, then computed crude rates of fracture outcomes per 1000 person-years. Analogous to the intention-to-treat approach used in RCTs,^26^ a patient was considered treated with the agent initiated on the index date. We included all patient data from the index date to the date of a fracture outcome, death, emigration, or December 31, 2017, whichever occurred first. Occurrence of a fracture at a site other than the hip did not censor follow-up for the hip fracture end point. We plotted cohort-specific cumulative incidence curves for hip fracture and any fracture, considering death as a competing risk and weighting all observations by stabilized inverse probability of treatment weights (IPTWs) to balance the measured covariates.^28^ Inverse probability of treatment weights were derived from propensity scores, which were computed using logistic regression as predicted probability of initiating denosumab or alendronate treatment as a function of the covariates and interaction terms of sex with comedication and of calendar year with all other covariates. Weights were stabilized by multiplying the weights in the denosumab cohort by the proportion of denosumab users in the total cohort and by multiplying the weights in the alendronate cohort by the proportion of alendronate users in the total cohort.^28^ We used standardized mean differences to assess the balance of covariates in the 2 cohorts, with the aim of achieving a standardized mean difference of less than 0.1 for all covariates.

Furthermore, we computed risk differences (based on cumulative incidences) with 95% CIs. We used Cox proportional hazards regression to compute crude and adjusted hazard ratios (aHRs) with 95% CIs for each outcome, overall and stratified by age group, sex, and fracture history. In the stratified analyses, new stabilized IPTWs were computed within each subgroup. The proportionality of hazards assumptions was found not to be violated, according to log-log plots.

We tested the robustness of the results in a series of sensitivity analyses. First, we repeated the analyses while excluding patients with a recent fracture at the same site as a given outcome, varying the definition of recent from 30 to 730 days before the index date. Second, we used a stricter definition of treatment initiation by extending the requirement of absence of pre–index date osteoporosis therapy from 1 year to 5 years. Third, we excluded atypical femoral fractures (approximated, in the absence of specific diagnostic codes for atypical fractures or access to results of radiography, by diagnostic codes for fractures of the femoral shaft or subtrochanteric region) from the hip fracture definition and computed new aHRs for hip fracture. Fourth, we repeated the analyses while censoring follow-up at discontinuation of or switch from the initial treatment (analogous to the per-protocol approach in RCTs). Discontinuation was defined as a gap of 90 days or more between the expiration of the days supplied in a given dispensing for the denosumab cohort and as a gap of 180 days or more for the alendronate cohort, thereby accounting for differences in the pharmacokinetic properties of the 2 agents (ie, faster clearance of denosumab).^10^ We tested different combinations of discontinuation-defining intervals for denosumab/alendronate: 90/90 days, 60/150 days, 120/210 days, and 120/180 days. A switch was defined as a dispensing of a different antiosteoporosis agent before discontinuation of the initial agent. Analyses were performed with SAS version 9.4 (SAS Institute Inc).

## Results

We identified 4624 initiators of denosumab and 87 731 initiators of alendronate. Of the 92 355 included patients, 75 046 (81.3%) were women, and the mean (SD) age was 71 (10) years. Table 1 shows patient characteristics in the 2 cohorts before applying IPTWs. There was a sharp increase in the number of users of alendronate in 2012. This increased prescription rate was because alendronate’s status changed from individual to general reimbursement. The denosumab cohort had a lower proportion of men than the alendronate cohort (12.7% [589] vs 19.0% [16 700]), while age distributions were similar in the 2 cohorts. In the denosumab cohort, 604 patients (13.1%) were aged 50 to 59 years, 1382 (29.9%) were 60 to 69 years, 1496 (32.4%) were 70 to 79 years, and 1142 (24.7%) were 80 years and older. In the alendronate cohorts, 12 344 of patients (14.1%) were aged 50 to 59 years, 26 923 (30.7%) were 60 to 69 years, 28 320 (32.3%) were 70 to 79 years, and 20 144 (23.0%) were 80 years and older. The denosumab cohort had higher prevalences of comorbidity and comedications than the alendronate cohort, including higher prevalence of renal impairment (3.5% [161] vs 1.3% [1131]). While standardized mean differences before applying IPTW were as high as 0.63 for some covariates, in the IPTW-stabilized population all standardized mean differences were less than 0.02 (Table 1). Median (interquartile range) follow-up time was 3.3 (1.5-5.3) years in the denosumab cohort and 3.1 (1.4-5.0) years in the alendronate cohort. The Figure shows cumulative incidences of hip fracture and any fracture during the follow-up time in the denosumab cohort and the alendronate cohort in the unweighted and IPTW populations.

**Table 1.** Baseline Characteristics of the Denosumab and Alendronate Cohorts in Denmark, 2010 to 2017

Characteristic	No. (%)			Standardized Mean Difference	
	Denosumab (n = 4624)	Alendronate (n = 87 731)	Total (N = 92 355)	Before Weighting	After Stabilized IPTW
Year					
2010	168 (3.6)	2856 (3.3)	3024 (3.3)	0.03	0
2011	940 (20.3)	4973 (5.7)	5913 (6.4)	0.63	0
2012	845 (18.3)	24 876 (28.4)	25 721 (27.9)	0.34	0.01
2013	583 (12.6)	13 641 (15.5)	14 224 (15.4)	0.12	0.01
2014	617 (13.3)	10 926 (12.5)	11 543 (12.5)	0.04	0.01
2015	582 (12.6)	10 495 (12.0)	11 077 (12.0)	0.03	0
2016	472 (10.2)	9489 (10.8)	9961 (10.8)	0.03	0
2017	417 (9.0)	10 475 (11.9)	10 892 (11.8)	0.14	0
Men	589 (12.7)	16 700 (19.0)	17 289 (18.7)	0.24	0
Age group, y					
50-59	604 (13.1)	12 344 (14.1)	12 948 (14.0)	0.04	0.01
60-69	1382 (29.9)	26 923 (30.7)	28 305 (30.6)	0.02	0.01
70-79	1496 (32.4)	28 320 (32.3)	29 816 (32.3)	0.00	0
≥80	1142 (24.7)	20 144 (23.0)	21 286 (23.0)	0.06	0
Charlson Comorbidity Index score					
0 (No comorbidity)	2741 (59.3)	53 787 (61.3)	56 528 (61.2)	0.06	0.01
1-2 (Moderate)	1519 (32.9)	28 671 (32.7)	30 190 (32.7)	0.01	0.01
≥3 (High)	364 (7.9)	5273 (6.0)	5637 (6.1)	0.10	0.01
Specific comorbidities					
History of fracture	1508 (32.6)	26 190 (29.9)	27 698 (30.0)	0.08	0
Chronic renal impairment	161 (3.5)	1131 (1.3)	1292 (1.4)	0.20	0
COPD	827 (17.9)	14 257 (16.3)	15 084 (16.3)	0.06	0
Alcohol-related disorders	118 (2.6)	2014 (2.3)	2132 (2.3)	0.02	0.02
Obesity	141 (3.0)	2613 (3.0)	2754 (3.0)	0.01	0.01
Comedication					
Corticosteroids	1699 (36.7)	36 153 (41.2)	37 852 (41.0)	0.13	0
Anticoagulants	1943 (42.0)	34 496 (39.3)	36 439 (39.5)	0.08	0
Hormone replacement therapy	1796 (38.8)	28 854 (32.9)	30 650 (33.2)	0.18	0.01
Antidepressants, antipsychotics, and sedatives	1799 (38.9)	28 820 (32.9)	30 619 (33.2)	0.18	0.01
Statins	1854 (40.1)	34 274 (39.1)	36 128 (39.1)	0.03	0.01
NSAIDs	3576 (77.3)	67 209 (76.6)	70 785 (76.6)	0.02	0.01
Antidiabetics	320 (6.9)	6985 (8.0)	7305 (7.9)	0.06	0.01
Antihypertensives	2513 (54.3)	46 095 (52.5)	48 608 (52.6)	0.05	0
Drugs for treatment of COPD	1501 (32.5)	25 978 (29.6)	27 479 (29.8)	0.09	0
Opioids	3124 (67.6)	53 044 (60.5)	56 168 (60.8)	0.21	0.01
Antithyroid drugs	170 (3.7)	2972 (3.4)	3142 (3.4)	0.02	0

Abbreviations: COPD, chronic obstructive pulmonary disease; IPTW, inverse probability of treatment weight; NSAIDs, nonsteroidal anti-inflammatory drugs.

**Figure.**
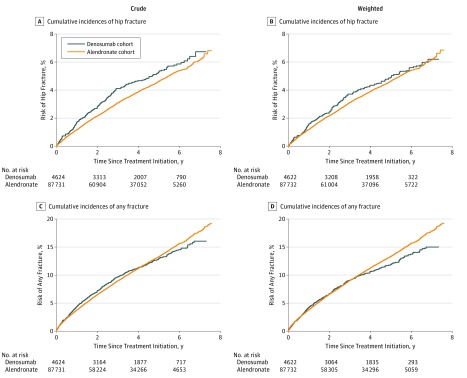
Crude and Weighted Cumulative Incidences of Hip Fracture and Any Fracture Following Initiation of Denosumab and Alendronate

### Hip Fracture

Within 3 years of follow-up, initiation of denosumab vs alendronate was associated with a weighted cumulative incidence for hip fracture of 3.7% vs 3.1%, respectively, corresponding to absolute risk differences of 0.6% (95% CI, −0.3% to 1.5%). The aHR for denosumab vs alendronate was 1.08 (95% CI, 0.92 to 1.28) for hip fracture (Table 2).

**Table 2.** Crude Rates and Crude and Adjusted Hazard Ratios of Hip Fracture in Denosumab and Alendronate Cohorts

Hip Fracture Outcome	Cohort	Patients, No.	Cases, No.	Person-Years	Crude Rate per 1000 Person-Years (95% CI)	Hazard Ratio (95% CI)	
						Crude	IPTW
Overall	Denosumab	4624	208	16 510.4	12.6 (10.9-14.4)	1.18 (1.03-1.36)	1.08 (0.92-1.28)
	Alendronate	87 731	3160	294 584.3	10.7 (10.4-11.1)	1 [Reference]	1 [Reference]
Age, y							
<80 y	Denosumab	3482	95	13 210.8	7.2 (5.8-8.8)	1.04 (0.84-1.28)	1.00 (0.78-1.28)
	Alendronate	67 587	1641	236 634.2	6.9 (6.6-7.3)	1 [Reference]	1 [Reference]
≥80 y	Denosumab	1142	113	3 299.6	34.2 (28.2-41.2)	1.30 (1.08-1.58)	1.21 (0.97-1.51)
	Alendronate	20 144	1519	57 950.1	26.2 (24.9-27.6)	1[Reference]	1 [Reference]
Sex							
Male	Denosumab	589	25	1745.3	14.3 (9.3-21.1)	1.38 (0.93-2.07)	1.24 (0.79-1.95)
	Alendronate	16 700	525	50 782.9	10.3 (9.5-11.3)	1 [Reference]	1 [Reference]
Female	Denosumab	4035	183	14 765.0	12.4 (10.7-14.3)	1.15 (0.99-1.34)	1.03 (0.87-1.22)
	Alendronate	71 031	2635	243 801.4	10.8 (10.4-11.2)	1 [Reference]	1 [Reference]
History of any fracture							
No	Denosumab	3116	106	11 477.1	9.2 (7.6-11.2)	1.17 (0.96-1.42)	1.05 (0.83-1.32)
	Alendronate	61 541	1673	212 601.9	7.9 (7.5-8.3)	1 [Reference]	1 [Reference]
Yes	Denosumab	1508	102	5033.2	20.3 (16.5-24.6)	1.13 (0.93-1.38)	1.07 (0.85-1.34)
	Alendronate	26 190	1487	81 982.4	18.1 (17.2-19.1)	1 [Reference]	1 [Reference]
History of hip fracture							
No	Denosumab	4139	161	15 122.8	10.6 (9.1-12.4)	1.15 (0.98-1.35)	1.04 (0.86-1.26)
	Alendronate	78 644	2496	268 782.3	9.3 (8.9-9.7)	1 [Reference]	1 [Reference]
Yes	Denosumab	485	47	1 387.5	33.9 (24.9-45.0)	1.33 (0.99-1.79)	1.25 (0.89-1.76)
	Alendronate	9087	664	25 802.0	25.7 (23.8-27.8)	1 [Reference]	1 [Reference]

Abbreviation: IPTW, inverse probability of treatment weight.

Hazard ratios for hip fracture were similar for denosumab vs alendronate, regardless of sex or age (Table 2). The aHR of denosumab vs alendronate for hip fracture was 1.07 (95% CI, 0.85 to 1.34) among patients with a history of any fracture and 1.05 (95% CI, 0.83 to 1.32) among patients without history of any fracture (Table 2). Weighted cumulative incidences for hip fracture within 3 years among patients with a history of any fracture were 6.1% and 5.1% in the denosumab cohort and the alendronate cohort, respectively, with an absolute risk difference of 1.0% (95% CI, −0.7 to 2.7). Weighted cumulative incidences of hip fracture within 3 years among patients without history of any fracture were 2.9% and 2.3% in the denosumab cohort and the alendronate cohort, respectively, with an absolute risk difference of 0.6% (95% CI, −0.3 to 1.5).

### Any Fracture

Within 3 years of follow-up, the initiation of denosumab and alendronate were both associated with a cumulative incidence of 9.0% for any fracture. The overall aHR for denosumab vs alendronate was 0.92 (95% CI, 0.83-1.02) for any fracture (Table 3). For denosumab vs alendronate, the aHR for any fracture was 0.84 (95% CI, 0.71-0.98) among patients with a history of any fracture and 0.77 (95% CI, 0.64-0.93) among patients without a history of any fracture. Results of stratified analyses for any fracture are presented in Table 3 and are very similar across strata.

**Table 3.** Crude Rates and Crude and Adjusted Hazard Ratios of Any Fracture in Denosumab and Alendronate Cohorts

Any Fracture Outcome	Cohort	Patients, No.	Cases, No.	Person-Years	Crude Rate per 1000 Person-Years (95% CI)	Hazard Ratio (95% CI)	
						Crude	IPTW
Overall	Denosumab	4624	511	15 749.0	32.4 (29.7-35.4)	0.99 (0.91-1.08)	0.92 (0.83-1.02)
	Alendronate	87 731	9213	280 839.0	32.8 (32.1-33.5)	1 [Reference]	1 [Reference]
Age							
<80 y	Denosumab	3482	313	12 655.1	24.7 (22.1-27.6)	0.93 (0.83-1.04)	0.85 (0.75-0.97)
	Alendronate	67 587	6052	226 136.1	26.8 (26.1-27.4)	1 [Reference]	1 [Reference]
≥80 y	Denosumab	1142	198	3093.9	64.0 (55.4-73.6)	1.10 (0.95-1.27)	1.06 (0.89-1.26)
	Alendronate	20 144	3161	54 702.9	57.8 (55.8-59.8)	1 [Reference]	1 [Reference]
Sex							
Male	Denosumab	589	46	1707.6	26.9 (19.7-35.9)	1.17 (0.87-1.57)	0.96 (0.68-1.36)
	Alendronate	16 700	1137	49 511.2	23.0 (21.6-24.3)	1[Reference]	1 [Reference]
Female	Denosumab	4035	465	14 041.4	33.1 (30.2-36.3)	0.95 (0.86-1.04)	0.89 (0.80-0.99)
	Alendronate	71 031	8076	231 327.8	34.9 (34.2-35.7)	[1 Reference]	1 [Reference]
History of any fracture							
No	Denosumab	1508	149	3357.7	44.4 (37.5-52.1)	0.82 (0.70-0.97)	0.77 (0.64-0.93)
	Alendronate	26 190	2556	45 211.4	56.5 (54.4-58.8)	1 [Reference]	1 [Reference]
Yes	Denosumab	1508	223	4722.2	47.2 (41.2-53.8)	0.89 (0.78-1.02)	0.84 (0.71-0.98)
	Alendronate	26 190	4054	76 222.9	53.2 (51.6-54.8)	1 [Reference]	1 [Reference]
History of hip fracture							
No	Denosumab	4139	428	14 452.6	29.6 (26.9-32.6)	0.98 (0.89-1.08)	0.89 (0.79-0.99)
	Alendronate	78 644	7778	256 616.5	30.3 (29.6-31.0)	1 [Reference]	1 [Reference]
Yes	Denosumab	485	83	1296.4	64.0 (51.0-79.4)	1.07 (0.86-1.34)	1.11 (0.85-1.44)
	Alendronate	9087	1435	24 222.5	59.2 (56.2-62.4)	1 [Reference]	1 [Reference]

Abbreviation: IPTW, inverse probability of treatment weight.

### Sensitivity Analyses

The results did not materially change after excluding patients with a recent fracture at the same site as a given outcome or after excluding atypical femoral fractures from the hip fracture outcome definition. Extending the required therapy-free period to 5 years to define new use yielded an aHR for hip fracture of 1.07 (95% CI, 0.88-1.30) and for any fracture of 0.93 (95% CI, 0.82-1.05) for denosumab compared with alendronate (eTable 1 in the Supplement).

Patients switching between treatments were rare. Discontinuation was more frequent; thus, we lost approximately 30% of person-years in the denosumab group and approximately 44% for the alendronate cohort (these figures were similar for hip and any fracture). Nevertheless, the on-treatment analyses were consistent with the findings for hip fracture in the primary analysis (eTable 2 in the Supplement).

## Discussion

This large nationwide historical cohort study was conducted using routinely and prospectively collected data originating from a health care system with universal coverage. Initiation of denosumab and initiation of alendronate were associated with similar risks of hip fracture or any fracture during a median follow-up of approximately 3 years, regardless of sex, age, or previous fracture.

Randomized clinical trials evaluating head-to-head efficacy of denosumab vs alendronate used BMD as the primary outcome and clinical fractures as an adverse effect or complication.^15,16^ These RCTs, conducted in postmenopausal women, regardless of prior treatment for osteoporosis, both found denosumab to be more efficacious than alendronate in increasing BMD, possibly the best proxy outcome for subsequent fracture risk. Overall, 6 of 8 RCTs^29,30,31,32,33,34^ included in 2 meta-analyses^15,16^ reported results on risk of any fracture. Our finding of no clinically relevant differences between denosumab and alendronate in the risk of hip and any fracture is in line with findings of the RCTs.^15,16^ In addition, our absolute risk estimates are similar to those from the RCTs; however, the absolute number of fractures in our study is considerably higher than the total number of fractures from RCTs included in the 2 meta-analyses.^15,16^ Kendler et al^30^ reported the risk of any fracture during 12 months of treatment as 3.2% (8 of 253 patients) in denosumab initiators and 1.6% (4 of 249 patients) in alendronate initiators. McClung et al^32^ reported 3.8% (12 of 314 patients) vs 2.2% (1 of 46 patients) risk of any fracture among individuals taking denosumab vs alendronate, respectively, and Brown et al^29^ reported 4.0% (24 of 593 patients) vs 3.2% (19 of 586 patients) risk of any fracture after 1 year among individuals taking denosumab vs alendronate. Two additional studies reported the risk of any fracture being 4.4% vs 3.5%^34^ and 3.6% vs 3.2%^33^ for denosumab users vs alendronate users, respectively, after 1 year of treatment, whereas a third study reported the risk of any fracture within 2 years of treatment as 6.7% vs 4.3%.^31^ To our knowledge, no head-to-head RCTs were powered to examine the risk of specific fractures, such as hip fractures, between denosumab and alendronate users. A 1996 meta-analysis based on women has suggested that the risk of hip fracture changes 2.6-fold for each standard deviation in BMD at the hip.^18^ The gradient of risk is lower (1.4 vs 1.6) in predicting any other osteoporotic fractures, regardless of the site measured.

It is well known that BMD can identify individuals who are at increased risk of developing a fracture, but it cannot with certainty identify individuals who will eventually develop fractures.^18^ Therefore, it is important to use data from clinical practice to examine treatment effectiveness in terms of fracture risk (clinically relevant outcome). Randomized clinical trials have not provided a clear answer on that question, especially as applied to long-term outcomes. In this study, it was not feasible to directly assess the full treatment-BMD-fracture pathway. However, BMD increase while receiving treatment accounts for only a small proportion of actual observed fracture risk reduction. A number of other factors, such as lifestyle factors, medication, comorbidities, or socioeconomic status, can influence the association of BMD with fracture risk and could explain why higher efficacy of denosumab on BMD does not translate into higher effectiveness in reducing hip fracture risk. In addition, we observed no difference in secondary hip fracture reduction between denosumab and alendronate treatment, which does not support current recommendations for prescribing denosumab to high-risk patients. The cost-effectiveness of denosumab treatment compared with alendronate is an argument for prescribing denosumab rather than alendronate to prevent hip fractures.

### Limitations

Our study had limitations. The most important limitation when comparing a new-in-class agent with an established treatment is the possibility of residual confounding. For example, renal impairment was more prevalent in the denosumab cohort than in the alendronate cohort but is incompletely measured by hospital diagnoses.^35^ We lacked measures of frailty, socioeconomic status, and lifestyle, which could affect our results. Furthermore, no data on BMD were available, which is likely the main driver of treatment choice. Most guidelines, including National Institute for Health and Care Excellence technology appraisal guideline 204 from 2010,^36^ recommend the use of denosumab only in patients with lower BMD or in secondary fracture prevention, if first-line alendronate is not tolerated or contraindicated. Thus, confounding by indication is another limitation of our study, despite comprehensive availability of confounders and use of stabilized IPTWs in statistical analyses. Also, despite the large sample size, it was not possible to divide patients into more than 2 age groups.

## Conclusions

In this nationwide cohort study based on routinely collected data in Denmark, treatment with denosumab and alendronate were associated with similar risks of hip and any fracture over a 3-year period. Sex, age, and fracture history were not associated with patients’ risk.

## Notes

Supplement. **eTable 1.** Rates and hazard ratios of the fracture outcomes in initiators of denosumab and alendronate, defining initiation by requiring 5 years’ absence of osteoporosis treatment before the index date (sensitivity analysis) **eTable 2.** Rates and hazard ratios of the fracture outcomes for denosumab and alendronate, with follow-up censored at discontinuation or switch
