# Supplementary material for: Comparison of Risk of Osteoporotic Fracture in Denosumab vs Alendronate Treatment Within 3 Years of Initiation
Source: JAMA Netw Open. 2019 Apr 19;2(4):e192416. doi: 10.1001/jamanetworkopen.2019.2416 (PMC6481596; doi:10.1001/jamanetworkopen.2019.2416)
Supplement: Supplement. — eTable 1. Rates and hazard ratios of the fracture outcomes in initiators of denosumab and alendronate, defining initiation by requiring 5 years’ absence of osteoporosis treatment before the index date (sensitivity analysis) eTable 2. Rates and hazard ratios of the fracture outcomes for denosumab and alendronate, with follow-up censored at discontinuation or switch [file jamanetwopen-2-e192416-s001.pdf]

Supplementary Online Content

Pedersen AB, Heide-Jørgensen U, Sørensen HT, Prieto-Alhambra D, Ehrenstein V. Comparison of risk of osteoporotic fracture in denosumab vs alendronate treatment within 3 years of initiation. *JAMA Netw Open.* 2019;2(4):e192416. doi:10.1001/jamanetworkopen.2019.2416

eTable 1. Rates and hazard ratios of the fracture outcomes in initiators of denosumab and alendronate, defining initiation by requiring 5 years' absence of osteoporosis treatment before the index date (sensitivity analysis)
eTable 2. Rates and hazard ratios of the fracture outcomes for denosumab and alendronate, with follow-up censored at discontinuation or switch

This supplementary material has been provided by the authors to give readers additional information about their work.

© 2019 Pedersen AB et al. *JAMA Network Open.*

eTable 1. Rates and hazard ratios of the fracture outcomes in initiators of denosumab and alendronate, defining initiation by requiring 5 years' absence of osteoporosis treatment before the index date (sensitivity analysis).

Analysis group	Cohort	N	Cases	PY[a]	Crude rate per 1,000 PY (95% CI)	Crude hazard ratio (95% CI)	IPTW[b] hazard ratio (95% CI)
Hip fracture	Denosumab	3,703	148	12,829.6	11.5 (9.8 - 13.6)	1.12 (0.95 - 1.32)	1.07 (0.88 - 1.30)
	Alendronate	83,110	2,841	274,546.6	10.3 (10.0 - 10.7)	Reference	Reference
Any fracture	Denosumab	3,703	374	12,274.5	30.5 (27.5 - 33.7)	0.95 (0.86 - 1.05)	0.93 (0.82 - 1.05)
	Alendronate	83,110	8,409	262,158.1	32.1 (31.4 - 32.8)	Reference	Reference

[a] PY- person years. [b] IPTW - inverse probability of treatment weight.

© 2019 Pedersen AB et al. *JAMA Network Open.*

eTable 2. Rates and hazard ratios of the fracture outcomes for denosumab and alendronate, with follow-up censored at discontinuation or switch.

Analysis group	Cohort	N	Cases	PY	Crude rate per 1,000 PY (95% CI)	Crude hazard ratio (95% CI)	IPTW hazard ratio (95% CI)
Hip fracture	Denosumab	4,624	122	11,633.2	10.5 (8.7 - 12.5)	1.01 (0.84 - 1.22)	0.88 (0.71 - 1.09)
	Alendronate	87,731	1,785	166,022.4	10.8 (10.3 - 11.3)	Reference	Reference
Any fracture	Denosumab	4,624	339	11,198.1	30.3 (27.1 - 33.7)	0.93 (0.83 - 1.03)	0.85 (0.75 - 0.97)
	Alendronate	87,731	5,438	160,218.3	33.9 (33.0 - 34.9)	Reference	Reference

[a] PY- person years. [b] IPTW - inverse probability of treatment weight.

© 2019 Pedersen AB et al. *JAMA Network Open.*
